# Involvement of the Hydroperoxy Group in the Irreversible Inhibition of Leukocyte-Type 12-Lipoxygenase by Monoterpene Glycosides Contained in the Qing Shan Lu Shui Tea

**DOI:** 10.3390/molecules24020304

**Published:** 2019-01-15

**Authors:** Yuki Kawakami, Akemi Otsuki, Yoshiko Mori, Keita Kanzaki, Toshiko Suzuki-Yamamoto, Ding Zhi Fang, Hideyuki Ito, Yoshitaka Takahashi

**Affiliations:** 1 Department of Nutritional Science, Faculty of Health and Welfare Science, Okayama Prefectural University, 111 Kuboki, Soja, Okayama 719-1197, Japan; kawaka@fhw.oka-pu.ac.jp (Y.K.); okapu.1222041akemi@gmail.com (A.O.); tyche55cbm@gmail.com (Y.M.); keita.kanzaki@mw.kawasaki-m.ac.jp (K.K.); toshiko@fhw.oka-pu.ac.jp (T.S.-Y.); hito@fhw.oka-pu.ac.jp (H.I.); 2 Department of Human Nutrition, Faculty of Contemporary Human Life Science, Chugoku Gakuen University, 83 Niwase, Kita-ku, Okayama 701-0197, Japan; 3 Department of Clinical Nutrition, Faculty of Health Science and Technology, Kawasaki University of Medical Welfare, 288 Matsushima, Kurashiki, Okayama 701-0193, Japan; 4 Department of Biochemistry and Molecular Biology, West China School of Preclinical and Forensic Medicine, Sichuan University, 17 Section 3, South Renmin Road, Chengdu 610041, China; dzfang@scu.edu.cn

**Keywords:** Qing Shan Lu Shui, *Ligustrum robustum*, liguroside, monoterpene glycoside, hydroperoxy group, leukocyte-type 12-lipoxygenase, irreversible inhibition

## Abstract

We have previously found two novel monoterpene glycosides, liguroside A and liguroside B, with an inhibitory effect on the catalytic activity of the enzyme leukocyte-type 12-lipoxygenase in the Qing Shan Lu Shui tea. Here, two new monoterpene glycosides, liguroside C and liguroside D which inhibit this enzyme, were isolated from the same tea. The spectral and chemical evidence characterized the structures of these compounds as (5*E*)-7-hydroperoxy-3,7-dimethyl-1,5-octadienyl-3-*O*-(α-l-rhamnopyranosyl)-(1′′→3′)-(4′′′-*O*-*trans*-*p*-coumaroyl)-β-d-glucopyranoside and (2*E*)-6-hydroxy-3,7-dimethyl-2,7-octadienyl-3-*O*-(α-l-rhamnopyranosyl)-(1′′→3′)-(4′′′-*O*-*trans*-*p*-coumaroyl)-β-d-glucopyranoside, respectively. These ligurosides, which irreversibly inhibited leukocyte-type 12-lipoxygenase, have a hydroperoxy group in the monoterpene moiety. Additionally, monoterpene glycosides had the same backbone structure but did not have a hydroperoxy group, such as kudingoside A and lipedoside B-III, contained in the tea did not inhibit the enzyme. When a hydroperoxy group in liguroside A was reduced by using triphenylphosphine, the resultant compound, kudingoside B, showed a lower inhibitory effect on the enzyme. These results strongly suggest the involvement of the hydroperoxy group in the irreversible inhibition of the catalytic activity of leukocyte-type 12-lipoxygenase by the monoterpene glycosides contained in the Qing Shan Lu Shui tea.

## 1. Introduction

**Figure 1:**
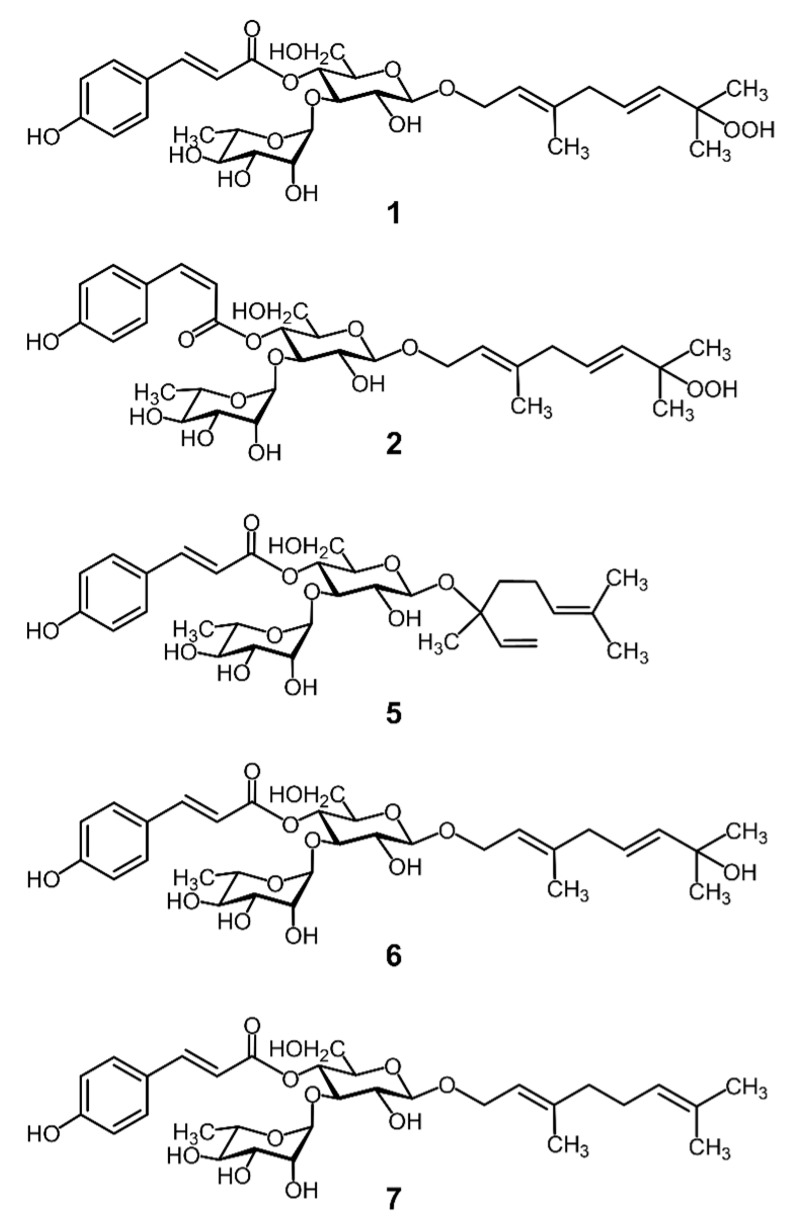
Structures of the known monoterpene glycosides; (**1**) liguroside A, (**2**) liguroside B, (**5**) lipedoside B-III, (**6**) kudingoside B, and (**7**) kudingoside A.

Qing Shan Lu Shui which is a kind of Kuding tea, is made by processing the leaves of *Ligustrum robustum* (Roxb.) Blume. The genus *Ligustrum* in the family Oleaceae contains phenolic constituents such as quinic acid derivatives, flavonoids, and monoterpene glycosides, and shows in vitro antioxidative activity [1]. Qing Shan Lu Shui tea is consumed in some parts of China, for health-benefits; it shows various pharmacological effects, such as anti-inflammatory, antioxidative, and antiproliferative activities [2,3,4,5,6]. A previous report has demonstrated that aqueous extracts of the processed leaves of *Ligustrum robustum*, dose-dependently scavenged superoxide radicals, inhibited lipid peroxidation, and prevented hemolysis of the red blood cells [2]. We have previously reported that two novel monoterpene glycosides, liguroside A and liguroside B, inhibited the catalytic activity of leukocyte-type 12-lipoxygenase (Figure 1) [7]. Leukocyte-type 12-lipoxygenase not only oxygenates the free fatty acids (such as arachidonic acid) to produce 12-hydroperoxy-5,8,10,14-eicosatetraenoic acid (12-HPETE), but also fatty acids that have esterified to cholesterol in the low-density lipoprotein (LDL) particle, which contributes to the formation of oxidized LDL and the development of atherosclerosis [8,9]. Our previous report demonstrated that guava leaf extracts inhibited the leukocyte-type 12-lipoxygenase activity, as well as cell-mediated LDL oxidation, and attenuated the development of atherosclerosis in apoE-knockout mice [10]. To further elucidate the beneficial components contained in the Qing Shan Lu Shui, we isolated novel monoterpene glycosides showing inhibitory effects on the leukocyte-type 12-lipoxygenase activity from the Qing Shan Lu Shui. We have also reported that the inhibitors from the Qing Shan Lu Shui irreversibly inhibit the catalytic activity of leukocyte-type 12-lipoxygenase.

## 2. Results and Discussion

In the present study, we isolated two new monoterpene glycosides, liguroside C and liguroside D, which inhibited the leukocyte-type 12-lipoxygenase from the Qing Shan Lu Shui tea.

**Figure 2:**
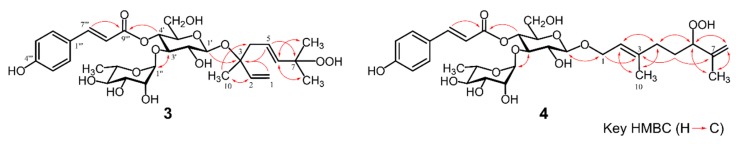
Structures of the new monoterpene glycosides, ligurosides C (**3**) and D (**4**).

Liguroside C, an off-white amorphous powder, exhibited an HRESIMS peak at *m*/*z* 639.2650 [M − H]^−^ (calcd. for C_31_H_43_O_14_, 639.2658). The ^1^H-NMR spectrum of liguroside A displayed signals due to a *trans p*-coumaroyl group [δ 7.51 (2H, d, *J* = 8.4 Hz), δ 6.85 (2H, d, *J* = 8.4 Hz), δ 7.64 (1H, d, *J* = 15.6 Hz), and 6.33 (1H, d, *J* = 15.6 Hz)], in the aromatic proton region. The existence of β-glucosyl and α-rhamnosyl moieties was deduced by two anomeric proton signals at δ 4.78 (d, *J* = 7.8 Hz) and δ 5.17 (brs), respectively. Proton signals due to an exomethylene (δ 5.19 and 5.18), three vinyls (δ 5.92, 5.63 and 5.59), a methylene (δ 2.32), and three tertiary methyl (δ 1.31, 1.22, 1.22) groups were observed in the aliphatic proton region (Table 1 and Figure S1). The ^13^C-NMR resonances of liguroside C were similar to those of liguroside A [7], except for 10 signals of a monoterpene moiety (Table 1 and Figures S2–S5). The monoterpene moiety was assigned as a (5*E*)-3,7-dimethyl-1,5-octadiene-3,7-diol analogue, based on the correlations from the HMBC experiment of liguroside C, as shown in Figure 2. Among the signals of monoterpene unit, the signals of the two methyls (δ 24.8, C-8, 9) and those attached to the oxygenated tertiary carbon (δ 81.6, C-7) were close to those due to the methyls (δ 24.1) and the methine (δ 80.6) carbons of liguroside A [7], suggesting that a hydroperoxy group was allocated to the C-7 position of liguroside C; this suggestion was supported by its high-resolution electrospray ionization mass spectroscopy (HRESIMS) analysis. On the basis of these data, the structure of liguroside C was concluded to be (5*E*)-7-hydroperoxy-3,7-dimethyl-1,5-octadienyl-3-*O*-(α-l-rhamnopyranosyl)-(1′′→3′)-(4′′′-*O*-*trans*-*p*-coumaroyl)-β-d-glucopyranoside (Figure 2).

Liguroside D was obtained as an off-white amorphous powder, which has the molecular formula C_31_H_44_O_14_, based on a pseudomolecular ion peak at *m*/*z* 639.2644 [M − H]^−^ (calcd. for C_31_H_43_O_14_, 639.2658) in the HRESIMS. The ^1^H- and ^13^C-NMR data corresponding to the acyl and glycosyl moieties of liguroside D were close to those of liguroside C, suggesting that an α-l-rhamnopyranosyl)-(1′′→3′)-(4′′′-*O*-*trans*-*p*-coumaroyl)-β-d-glucopyranosyl unit is present in liguroside D. The ^1^H- and ^13^C-NMR data of liguroside D, except for the signals due to the acyl and glycosyl units, showed the signals of an exomethylene (δ_H_ 4.89, 4.90; δ_C_ 113.7), a vinyl (δ_H_ 5.35; δ_C_ 121.4), oxygenated methine (δ_H_ 4.21; δ_C_ 88.8) and methylene (δ_H_ 4.32, 4.22; δ_C_ 65.8), mutually coupled methylenes (δ_H_ 2.03, 1.64, 1.56; δ_C_ 36.0, 29.5), two vinyl methyls (δ_H_ 1.67, 1.65; δ_C_ 17.0, 16.3), and two *sp*^2^ carbons (δ_C_ 145.4, 140.8) (Table 1 and Figures S6–S9). The NMR data of the monoterpene unit (Table 1) and the HMBC experiment of liguroside D (Figure 2 and Figure S10) suggested that the (2*E*)-6-hydroxy-3,7-dimethyl-2,7-octadien-1-ol analogue was involved in liguroside D, as a monoterpene unit. The significant downfield shift of the oxygenated methine resonance (δ_C_ 88.8) and HRESIMS data of liguroside D showed that the hydroxy group of C-6 position in liguroside D is replaced by the hydroperoxy group [11]. The NMR feature including the HMBC correlations of liguroside D confirmed that the structure of liguroside D was assigned to (2*E*)-6-hydroxy-3,7-dimethyl-2,7-octadienyl-3-*O*-(α-l-rhamnopyranosyl)-(1′′→3′)-(4′′′-*O*-*trans*-*p*-coumaroyl)-β-d-glucopyranoside (Figure 2).

**Figure 3:**
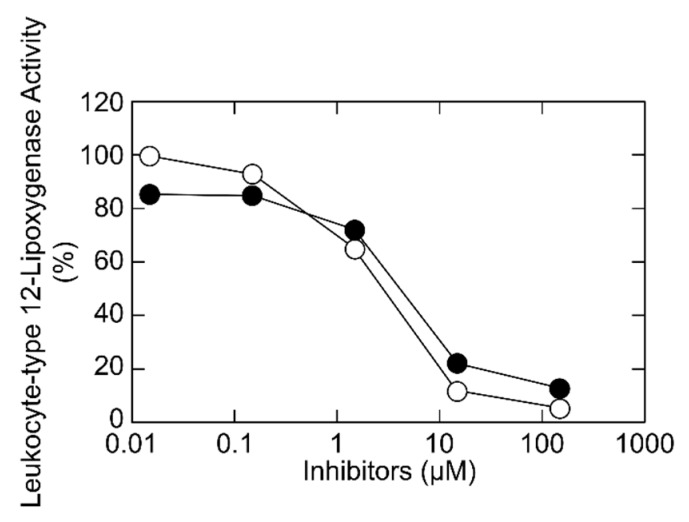
Inhibition of the leukocyte-type 12-lipoxygenase activity by liguroside C (closed circles) and liguroside D (open circles). Leukocyte-type 12-lipoxygenase was incubated with 25 μM arachidonic acid at 30 °C for 5 min in the standard reaction mixture, in the presence of inhibitors at various concentrations and the products were quantified using a reverse-phase HPLC. Relative enzyme activities, as compared with the activity without inhibitors, are shown.

As shown in Figure 3, liguroside C and liguroside D, dose-dependently inhibited the leukocyte-type 12-lipoxygenase activity with IC_50_ values of 2.9 μM and 4.2 μM, respectively. The inhibitory potencies of liguroside C and liguroside D were comparable to those of liguroside A and liguroside B (IC_50_ values of 1.7 μM and 0.7 μM) [7]. In our previous study, the leukocyte-type 12-lipoxygenase inhibitory assay-guided fractionation of the 30% aqueous ethanol soluble portion of the ethyl acetate extract was carried out using a reversed-phase HPLC [7]. We analyzed the obtained active fraction containing ligurosides A, B, C, and D using a reversed-phase HPLC (Figure S11A). Ligurosides C and D were separated by another HPLC condition (Figure S11B). Our previous report indicated that the contents of ligurosides A and B in the Qing Shan Lu Shui leaves were 0.85% and 0.31% (*w*/*w*), respectively [7]. The contents of ligurosides C and D were 0.23% and 0.50% (*w*/*w*), respectively.

**Figure 4:**
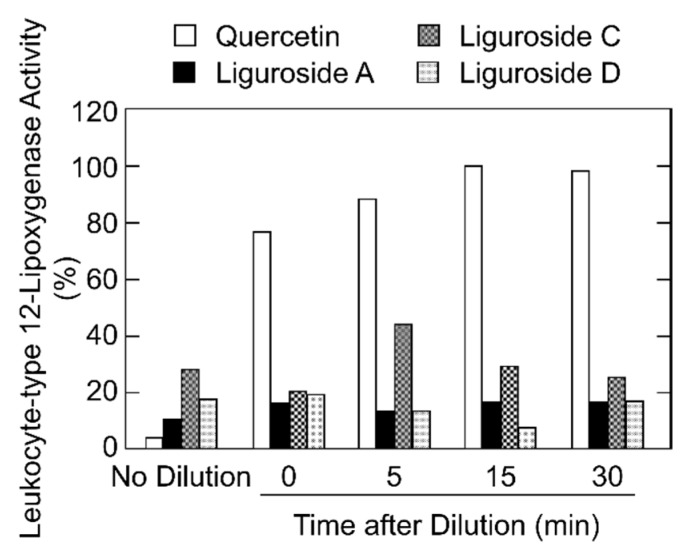
Reversibility analysis of the leukocyte-type 12-lipoxygenase inhibition. The leukocyte-type 12-lipoxygenase was preincubated with 6 μM quercetin, liguroside A, liguroside C, and liguroside D, at 30 °C, for 5 min, and then the mixture was 200-fold diluted in the standard lipoxygenase reaction mixture. After 0, 5, 15, and 30 min, the mixtures were incubated with 25 μM arachidonic acid at 30 °C, for 5 min. The reaction without dilution of inhibitors (no dilution) was also carried out. The relative enzyme activities, as compared with the activity without inhibitors, are shown.

Leukocyte-type 12-lipoxygenase shows an irreversible suicidal inactivation in which the reaction ceases within a few minutes resulting in a by-product of 15-hydroperoxy-5,8,11,13-eicosatetraenoic acid [12,13]. As all four compounds which inhibited leukocyte-type 12-lipoxygenase had a hydroperoxy group in the monoterpene moiety, we tested the reversibility of the leukocyte-type 12-lipoxygenase inhibition by ligurosides. The leukocyte-type 12-lipoxygenase was preincubated for 5 min with 6 μM of each liguroside or quercetin (used as a control of reversible inhibition of the enzyme), either of which almost completely inhibited the enzyme activity. The preincubation mixture was 200-fold diluted 0, 5, 15, and 30 min, before starting the enzyme reaction under the standard condition for the lipoxygenase assay. As shown in Figure 4, the leukocyte-type 12-lipoxygenase activity was quickly recovered after dilution of quercetin and the inhibition was hardly observed after 15 min. On the other hands, tested ligurosides inhibited the leukocyte-type 12-lipoxygenase activity, even after 30 min, indicating an irreversible inhibition of the enzyme activity (Figure 4).

**Figure 5:**
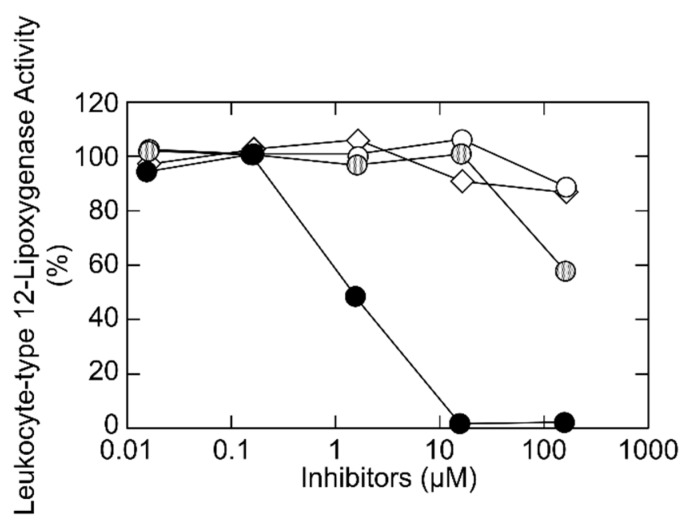
Inhibition of the leukocyte-type 12-lipoxygenase activity by liguroside A (closed circles), kudingoside B (grey circles), kudingoside A (open circles), and lipedoside B-III (open lozenges). Leukocyte-type 12-lipoxygenase was incubated with 25 μM arachidonic acid, at 30 °C, for 5 min, in the standard reaction mixture in the presence of inhibitors, at various concentrations, and the products were quantified using a reverse-phase HPLC. Relative enzyme activities, as compared with the activity without inhibitors, are shown.

*p*-Coumaric acid, as a part of the ligurosides, showed no inhibition on the leukocyte-type 12-lipoxygenase activity, suggesting that the inhibitory effects of liguroside A and liguroside B can be attributed to the monoterpene unit with the hydroperoxy group [7]. On the other hand, monoterpene glycosides have the same backbone structure but did not have a hydroperoxy group (such as kudingoside A and lipedoside B-III) contained in the tea (Figure 1), and did not inhibit the enzyme activity (Figure 5). We reduced a hydroperoxy group in liguroside A using triphenylphosphine. The structure of the reduced compound was confirmed by mass spectrometry as kudingoside B, as described previously (Figure 1) [7]. The resultant compound, kudingoside B, showed a lower inhibitory effect on the enzyme (Figure 5), strongly suggesting that the hydroperoxy group of ligurosides played a key role in the irreversible inhibition of the leukocyte-type 12-lipoxygenase by the compounds. The detailed mechanism of the enzyme inhibition by the hydroperoxy group of ligurosides remains to be elucidated. In the suicidal inactivation of the enzyme, 15-hydroperoxy-5,8,11,13-eicosatetraenoic acid was further converted to 14,15-leukotriene A_4_, an epoxide product, by the leukocyte-type 12-lipoxygenase, itself, and was incorporated into the enzyme protein, by a covalent binding [12]. In contrast, 12-hydroperoxy-5,8,10,14-eicosatetraenoic acid, a major product of the leukocyte-type 12-lipoxyganase, did not inactivate the enzyme as fast as the 15-hydroperoxy-5,8,11,13-eicosatetraenoic acid, and slowly incorporated into the enzyme that did not bind covalently [12]. It is well-known that terpenes, such as linalool and geraniol can autoxidize and form oxidation products such as hydroperoxides, when exposed to air [14]. It was also reported that the hydroxy-group-esterified linalyl acetate oxidizes on air-exposure, forming hydroperoxides, which then turns into epoxide [15]. Therefore, the liguroside epoxides derived from the hydroperoxy group of the ligurosides might contribute to the irreversible inhibition of the leukocyte-type 12-lipoxygenase.

**Figure 6:**
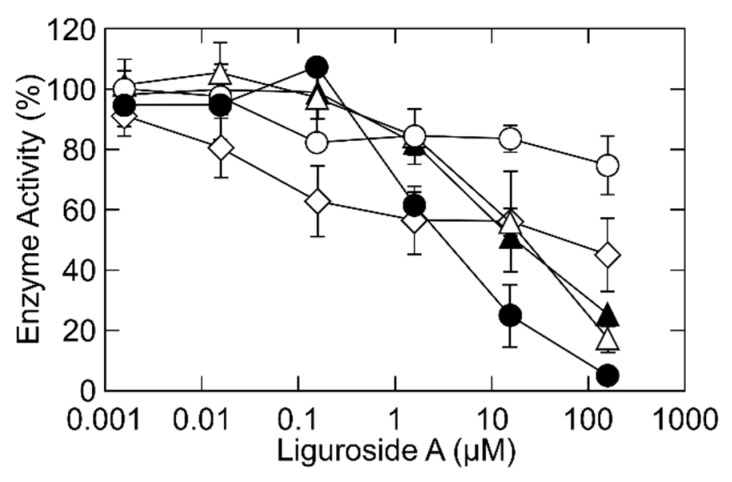
Inhibition of various lipoxygenase and cyclooxygenase activities by liguroside A. Reactions were carried out under the standard conditions in the presence of liguroside A at various concentrations and the products were quantified, using a reverse-phase HPLC. Relative enzyme activities of the 15-lipoxygenase-1 (closed circles), platelet-type 12-lipoxygenase (open circles), 5-lipoxygenase (open lozenges), cyclooxygenase-1 (open triangles), and cyclooxygenase-2 (closed triangles), as compared to the activity without inhibitors, are shown.

We investigated whether the ligurosides affect the catalytic activities of the arachidonic acid, metabolizing enzymes other than the leukocyte-type 12-lipoxygenase. As shown in Figure 6, liguroside A also inhibited 15-lipoxygenase, cyclooxygenase-1, and cyclooxygenase-2, with IC_50_ values of 3.2, 21.5, and 16.9 μM, respectively. In contrast, platelet-type 12-lipoxygenase was almost unaffected by liguroside A up to 100 μM. Kishimoto et al. reported that the platelet-type 12-lipoxygenase did not show a rapid inactivation by the 15-hydroperoxy-5,8,11,13-eicosatetraenoic acid, presumably because the enzyme hardly converted the 15-hydroperoxy-5,8,11,13-eicosatetraenoic acid to an epoxide product [12]. It is known that suicidal inactivation is hardly observed in the platelet-type 12-lipoxygenase that did not produce the 15-hydroperoxy-5,8,11,13-eicosatetraenoic acid as a by-product [12]. Further investigation is necessary to elucidate the mechanism of inhibition of the leukocyte-type 12-lipoxygenase by the ligurosides.

## 3. Materials and Methods

### 3.1. Materials

Arachidonic acid was obtained from the Nu-Chek prep (Elysian, MN, USA). Authentic 15-hydroxy-11,13-eicosadienoic acid (15-HEDE) was prepared by the incubation of soybean lipoxygenase type I (Sigma-Aldrich, St. Louis, MO, USA), with 11,14-eicosadienoic acid (Cayman Chemical, Ann Arbor, MI, USA), followed by purification with HPLC [9]. Kudingoside B was prepared by the incubation of liguroside A, with triphenylphosphine, as previously described [7]. All other reagents and chemicals were commercially available as extra-pure-grade products.

### 3.2. Isolation of the Monoterpene Glycosides

The aqueous ethanol extract of the Qing Shan Lu Shui was prepared, as described previously [7]. Briefly, the dried leaves of the Qing Shan Lu Shui were extracted with 50% aqueous ethanol. After filtration and evaporation of the extract, the residue was partitioned between water and ethyl acetate. The organic layer was evaporated, and then, the resulting residue was re-dissolved with 45% aqueous methanol or 53% aqueous methanol. The 45% aqueous methanol extract was separated by a preparative HPLC, to afford liguroside C (retention time 31.5 min) and liguroside D (retention time was 37.6 min). HPLC was performed on a Waters Alliance 2695 separations module. Reverse-phase HPLC in an isocratic condition was conducted on a COSMOSIL Cholester column (5-μm particle, 250 × 4.6 mm i.d., Nacalai, Kyoto, Japan), developed with methanol–water–acetic acid (45:55:0.01, *v*/*v*), at 40 °C and a flow rate of 1.0 mL/min. Detection was affected at 200–700 nm. The 53% aqueous methanol extract was separated by a preparative HPLC to afford the kudingoside A (retention time 31.5 min) and lipedoside B-III (retention time 37.6 min). HPLC was performed under the same conditions as those described above, but was not developed with methanol-ater-acetic acid (53:47:0.01, *v*/*v*).

### 3.3. Structural Analysis

^1^H- and ^13^C-NMR spectra were measured in acetone-*d*_6_ on a Varian NMR System 600 MHz (600 MHz for ^1^H-NMR and 151 MHz for ^13^C-NMR) instrument; chemical shifts were given in δ (ppm) values, relative to that of the solvent (δ_H_: 2.04; δ_C_: 29.8), on a tetramethylsilane scale. The standard pulse sequences programmed for the instrument were used for each 2D-NMR experiment (^1^H-^1^H COSY, HSQC, and HMBC). The *J*_CH_ value was set at 8 Hz in the HMBC experiment. Mass spectra were obtained on a Bruker MicrOTOF II spectrometer (Bruker, Billerica, MA, USA), using ESI source in a negative-ion mode.

### 3.4. Enzyme Assay

The lipoxygenase and cyclooxygenase reactions were carried out, as described previously [9,10,16]. Briefly, leukocyte-type 12-lipoxygenase or 15-lipoxygenase-1 was preincubated with inhibitors, in a volume of 0.2 mL in 100 mM Tris-HCl buffer, at pH 7.4, for 5 min at 30 °C, and was then incubated with 25 μM arachidonic acid for 5 min at 30 °C. Platelet-type 12-lipoxygenase reaction was carried out the as same as leukocyte-type 12-lipoxygenase reaction, except for incubation condition of 30 min at 37 °C. 5-Lipoxygenase was preincubated in 100 mM Tris-HCl buffer at pH 7.4, 2 mM CaCl_2_, and 2 mM ATP, with inhibitors, for 5 min on ice and then incubated with 25 μM arachidonic acid, for 5 min at 30 °C. To reduce the metabolites of each lipoxygenase isozyme, we added glutathione peroxidase (0.1 unit) and 5 mM glutathione to the mixture, followed by incubation for another 20 min. The enzyme reactions of cyclooxygenase-1 and cyclooxygenase-2 were carried out, as described previously [10,17]. Briefly, cyclooxygenase-1 and cyclooxygenase-2 were preincubated with inhibitors in a volume of 0.2 mL in 100 mM Tris-HCl buffer at pH 7.4, 2 μM hematin, and 5 mM tryptophan, for 5 min at 24 °C, and then incubated with 25 μM linoleic acid, for 5 min at 24 °C. After the reaction was quenched by an addition of 50 mM HCl, 0.5 nmol of 15-HEDE were added as an internal standard for enzyme reactions. The products extracted with ice-cold diethyl ether were analyzed by a reverse-phase HPLC, using a Waters Alliance system equipped with a COSMOSIL 5C18-MS-II column (5-μm particle, 250 × 4.6 mm i.d., Nacalai), with a solvent system of methanol-water-acetic acid (80:20:0.01, *v*/*v*), at a flow rate of 1 mL/min, as previously described [17]. Absorption at 235 nm was continuously monitored, using a Waters 2489 UV/Visible detector (Waters, Milford, MA, USA). Protein concentration was determined using a bicinchoninic acid protein assay kit (Thermo Fisher Scientific, Waltham, MA, USA), with bovine serum albumin as a standard.

## Figures and Tables

**Table 1:** ^1^H- and ^13^C-NMR data of ligurosides C and D in acetone-*d*_6_-D_2_O (9:1, *v*/*v*) (δ_H_: 600 MHz; δ_C_: 151 MHz).

Position	Liguroside C		Liguroside D	
	δ_H_ (*J* in Hz)	δ_C_	δ_H_ (*J* in Hz)	δ_C_
Monoterpene				
1	5.19 brd (18)	115.9	4.22 m	65.8
	5.18 brd (10.8)		4.32 dd (6, 12)	
2	5.92 dd (10.8, 18)	143.3	5.35 brt (6.6)	121.4
3		80.7		140.8
4	2.32 d (6)	44.7	2.03 m	36.0
5	5.63 dd (6, 15.6)	125.8	1.54 m, 1.64 m	29.5
6	5.59 d (15.6)	138.5	4.21 m	88.8
7		81.6		145.4
8	1.22 s	24.8	4.89 brs, 4.90 brs	113.7
9	1.22 s	24.8	1.67 s	17.0
10	1.31 s	23.1	1.65 s	16.3
Glucosyl				
1′	4.78 d (7.8)	98.6	4.41 d (7.8)	101.9
2′	3.38 brt (9)	75.5	3.40 brt (9)	75.5
3′	3.78 t (9)	80.8	3.81 t (9)	80.8
4′	4.85 t (9)	70.2	4.86 t (9)	70.2
5′	3.52 m	75.2	3.52 m	75.5
6′	3.54 m, 3.46 m	61.8	3.56 m, 3.49 m	61.9
Rhamnosyl				
1′′	5.17 brs	102.1	5.17 brs	102.2
2′′	3.90 m	71.5	3.90 m	71.5
3′′	3.54 m	71.6	3.54 m	71.6
4′′	3.29 t (9.6)	72.9	3.29 t (9.6)	73.0
5′′	3.51 m	69.6	3.51 m	69.6
6′′	1.02 d (6.6)	18.3	1.02 d (6.6)	18.3
Coumaroyl				
1′′′		126.3		126.4
2′′′	7.51 d (8.4)	131.0	7.51 d (8.4)	131.0
3′′′	6.85 d (8.4)	116.6	6.86 d (8.4)	116.6
4′′′		160.8		160.8
5′′′	6.85 d (8.4)	116.6	6.86 d (8.4)	116.6
6′′′	7.51 d (8.4)	131.0	7.51 d (8.4)	131.0
7′′′	7.64 d (15.6)	146.7	7.63 d (15.6)	146.7
8′′′	6.33 d (15.6)	114.5	6.33 d (15.6)	114.5
9′′′		167.5		167.5

## Supplementary Materials

The following are available online, Figure S1: ^1^H-NMR spectrum of liguroside C in acetone-*d*_6_-D_2_O (9:1, *v*/*v*) (600 MHz); Figure S2: ^13^C-NMR spectrum of liguroside C in acetone-*d*_6_-D_2_O (9:1, *v*/*v*) (151 MHz); Figure S3: ^1^H-^1^H COSY NMR spectrum of liguroside C in acetone-*d*_6_-D_2_O (9:1, *v*/*v*) (600 MHz); Figure S4: HSQC NMR spectrum of liguroside C in acetone-*d*_6_-D_2_O (9:1, *v*/*v*) (600 MHz); Figure S5: HMBC NMR spectrum of liguroside C in acetone-*d*_6_-D_2_O (9:1, *v*/*v*) (600 MHz); Figure S6: ^1^H-NMR spectrum of liguroside D in acetone-*d*_6_-D_2_O (9:1, *v*/*v*) (600 MHz); Figure S7: ^13^C-NMR spectrum of liguroside D in acetone-*d*_6_-D_2_O (9:1, *v*/*v*) (151 MHz); Figure S8: ^1^H-^1^H COSY NMR spectrum of liguroside D in acetone-*d*_6_-D_2_O (9:1, *v*/*v*) (600 MHz); Figure S9: HSQC NMR spectrum of liguroside D in acetone-*d*_6_-D_2_O (9:1, *v*/*v*) (600 MHz); Figure S10: HMBC NMR spectrum of liguroside D in acetone-*d*_6_-D_2_O (9:1, *v*/*v*) (600 MHz); Figure S11: HPLC profiles of ligurosides A, B, C, and D.
