# Supplementary material for: Involvement of the Hydroperoxy Group in the Irreversible Inhibition of Leukocyte-Type 12-Lipoxygenase by Monoterpene Glycosides Contained in the Qing Shan Lu Shui Tea
Source: Molecules. 2019 Jan 15;24(2):304. doi: 10.3390/molecules24020304 (PMC6358863; doi:10.3390/molecules24020304)
Supplement: Supplementary file 1 [file molecules-24-00304-s001.pdf]

Supplemental Information

Involvement of the Hydroperoxy Group in the Irreversible Inhibition of Leukocyte-Type 12-Lipoxygenase by Monoterpene Glycosides Contained in the Qing Shan Lu Shui Tea

Yuki Kawakami [1], Akemi Otsuki [1], Yoshiko Mori [1,2], Keita Kanzaki [1,3], Toshiko Suzuki-Yamamoto [1], Ding Zhi Fang [4], Hideyuki Ito [1] and Yoshitaka Takahashi [1,*]

[1] Department of Nutritional Science, Faculty of Health and Welfare Science, Okayama Prefectural University, 111 Kuboki, Soja, Okayama 719-1197, Japan; kawaka@fhw.oka-pu.ac.jp (Y.K.); okapu.1222041akemi@gmail.com (A.O.); tyche55cbm@gmail.com (Y.M.); keita.kanzaki@mw.kawasaki-m.ac.jp (K.K.); toshiko@fhw.oka-pu.ac.jp (T.S.-Y.); hito@fhw.oka-pu.ac.jp (H.I.)

[2] Department of Human Nutrition, Faculty of Contemporary Human Life Science, Chugoku Gakuen University, 83 Niwase, Kita-ku, Okayama 701-0197, Japan

[3] Department of Clinical Nutrition, Faculty of Health Science and Technology, Kawasaki University of Medical Welfare, 288 Matsushima, Kurashiki, Okayama 701-0193, Japan

[4] Department of Biochemistry and Molecular Biology, West China School of Preclinical and Forensic Medicine, Sichuan University, 17 Section 3, South Renmin Road, Chengdu 610041, China; dzfang@scu.edu.cn

* Correspondence: ytaka@ fhw.oka-pu.ac.jp; Tel.: +81-866-94-2155

Supplementary Figures

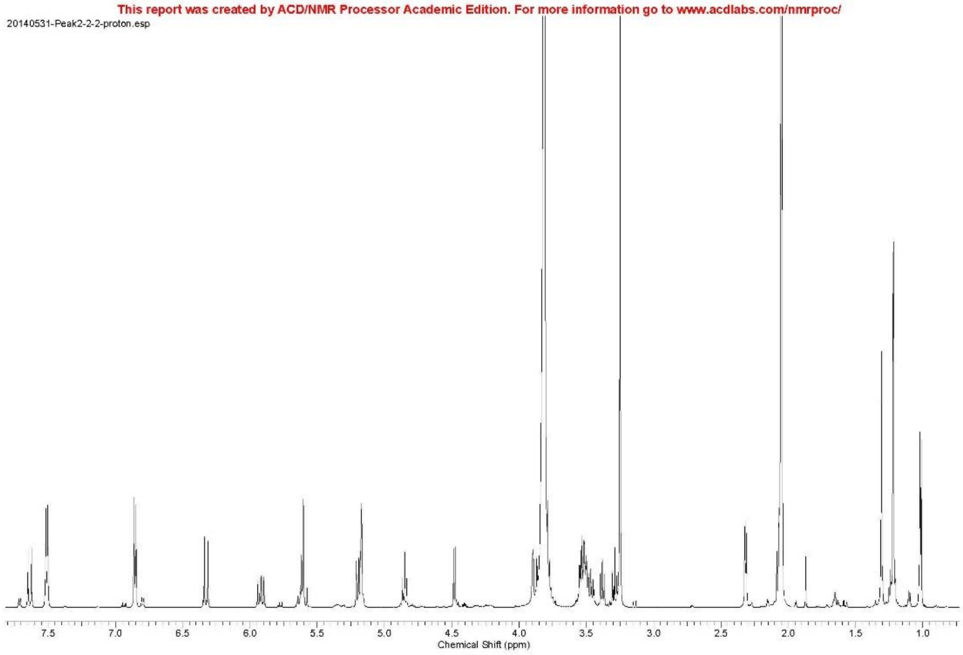

Figure S1. ^{1}H-NMR spectrum of liguroside C in acetone-d_6-D_2O (9:1, v/v) (600 MHz).

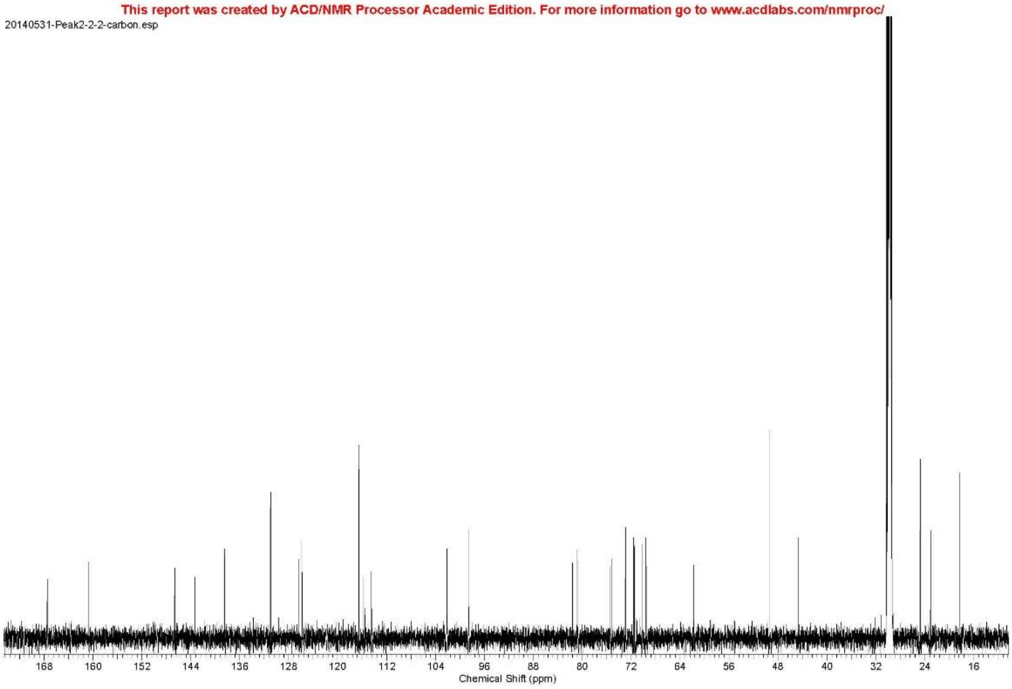

Figure S2. ^{13}C-NMR spectrum of liguroside C in acetone-d_6-D_2O (9:1, v/v) (151 MHz).

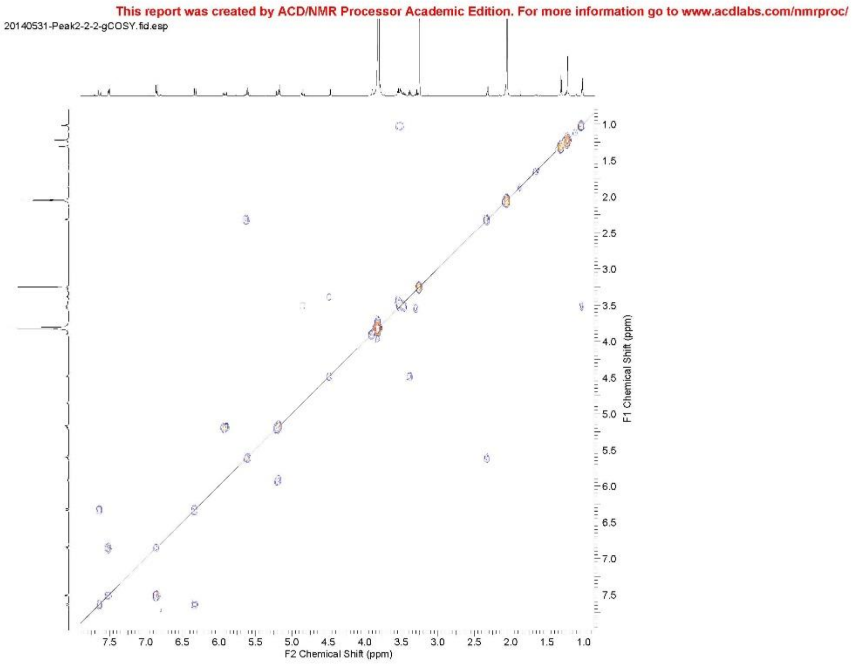

Figure S3. ^{1}H-^{1}H COSY NMR spectrum of liguroside C in acetone-d_6-D_2O (9:1, v/v) (600 MHz).

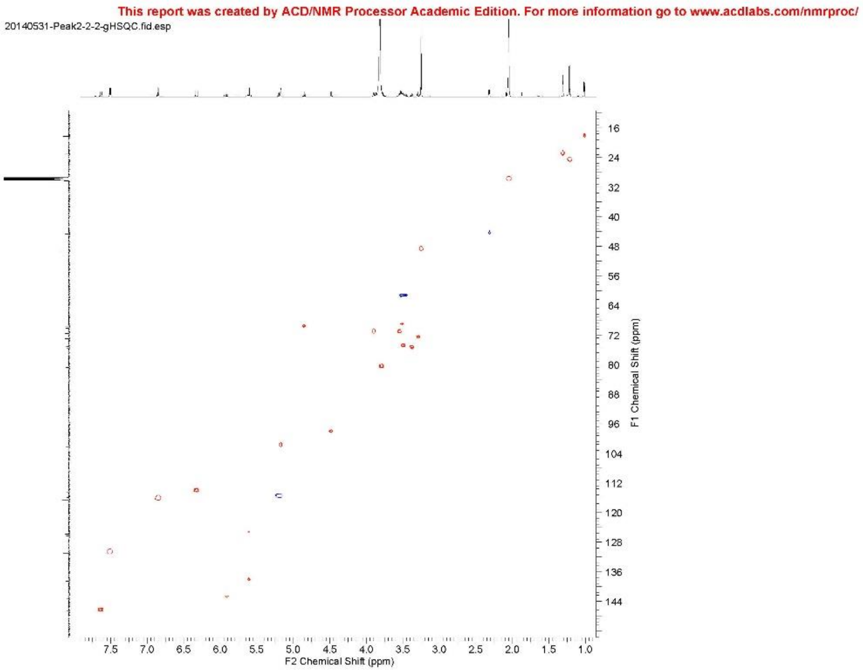

Figure S4. HSQC NMR spectrum of liguroside C in acetone-d_6-D_2O (9:1, v/v) (600 MHz).

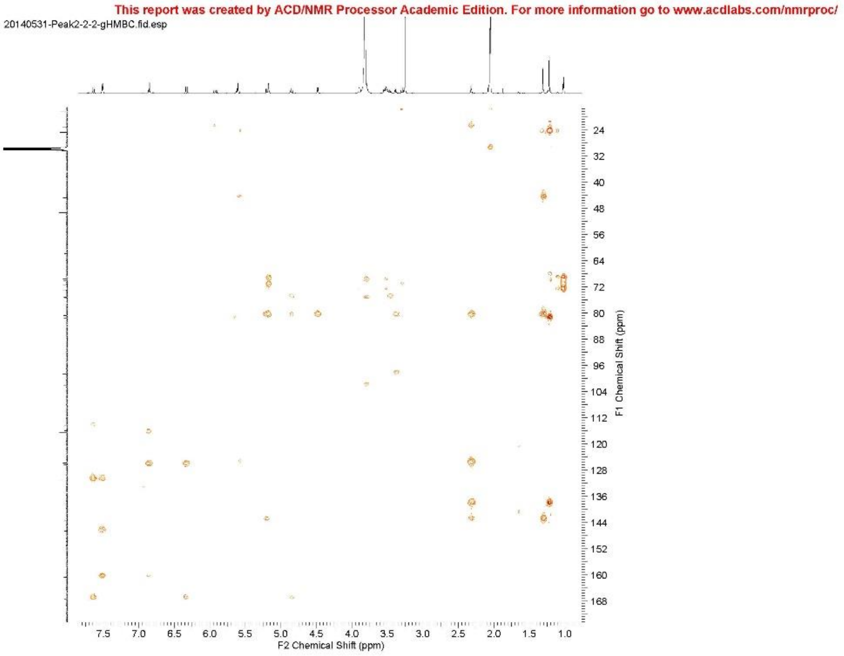

Figure S5. HMBC NMR spectrum of liguroside C in acetone-d_6-D_2O (9:1, v/v) (600 MHz).

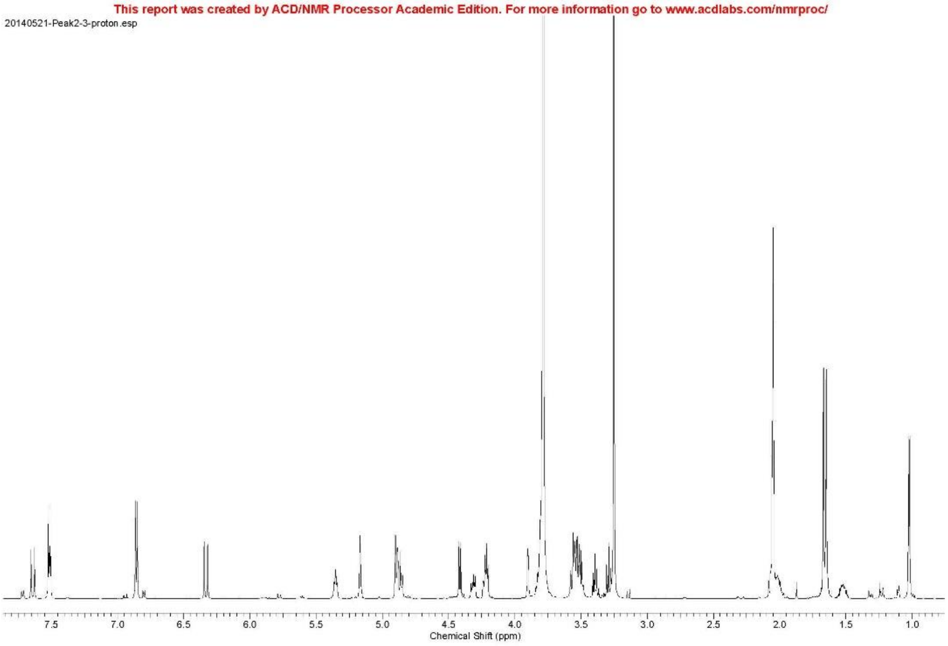

Figure S6. ^{1}H-NMR spectrum of liguroside D in acetone-d_6-D_2O (9:1, v/v) (600 MHz).

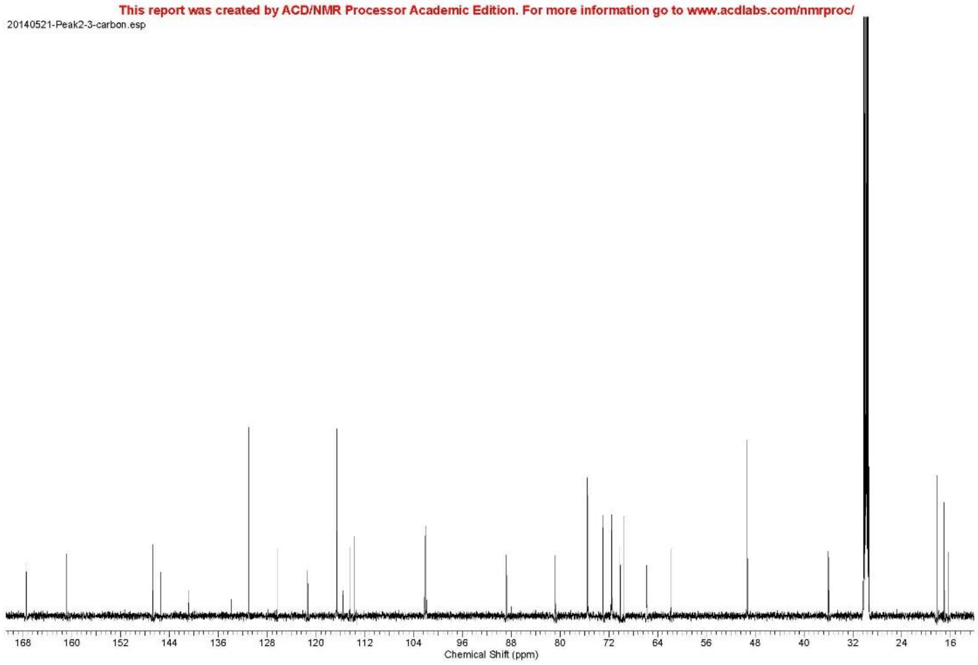

Figure S7. ^{13}C-NMR spectrum of liguroside D in acetone-d_6-D_2O (9:1, v/v) (151 MHz).

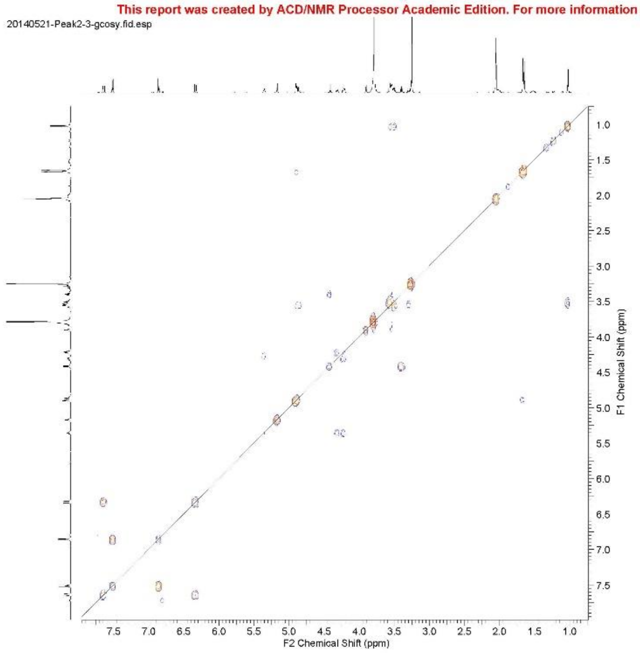

Figure S8. ^{1}H-^{1}H COSY NMR spectrum of liguroside D in acetone-d_6-D_2O (9:1, v/v) (600 MHz).

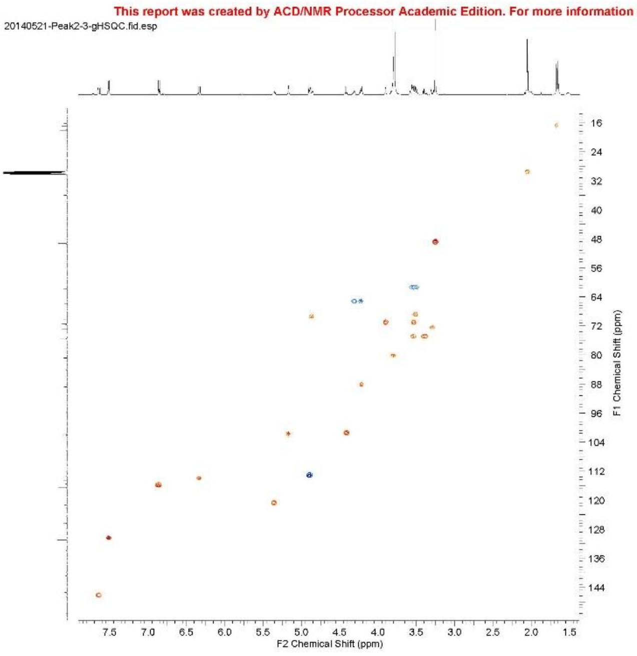

Figure S9. HSQC NMR spectrum of liguroside D in acetone-d_6-D_2O (9:1, v/v) (600 MHz).

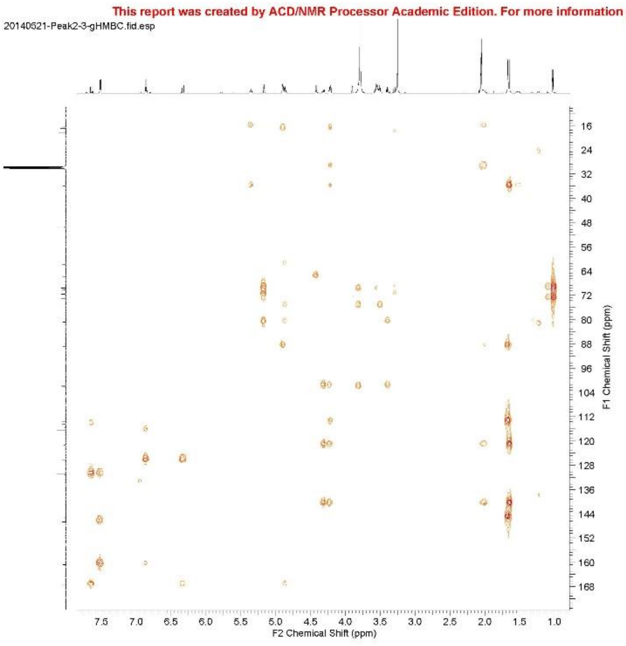

Figure S10. HMBC NMR spectrum of liguroside D in acetone-d_6-D_2O (9:1, v/v) (600 MHz).

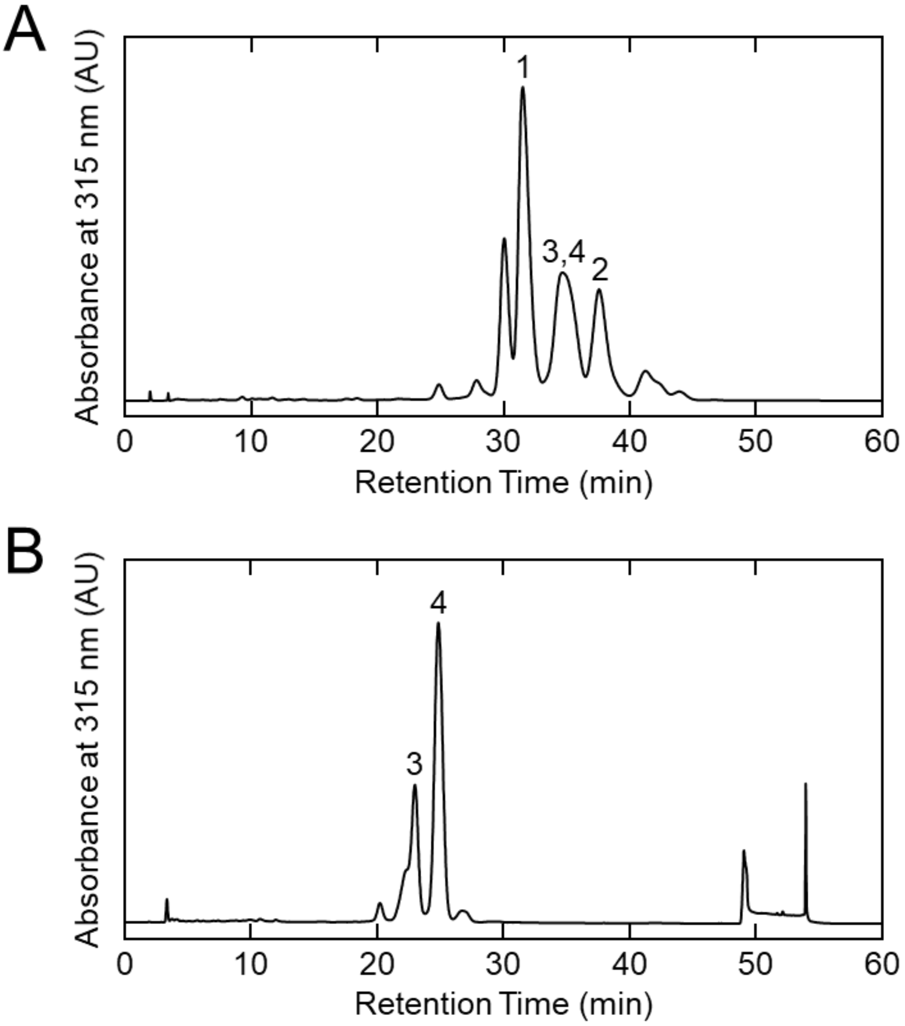

Figure S11. HPLC profiles of ligurosides A, B, C, and D. **(A)**, HPLC was performed on a Waters Alliance 2695 separations module. Reverse-phase HPLC in an isocratic condition was conducted on an Inertsil ODS-3 column (5-µm particle, 250 × 4.6 mm i.d., GL Sciences, Tokyo, Japan) developed with methanol–water–acetic acid (47:53:0.01, v/v) at 40 °C and a flow rate of 1.0 mL/min. Detection was effected at 200–700 nm. **(B)**, HPLC was performed under the same conditions as those described above, except for conducted on a COSMOSIL Cholester column (5-µm particle, 250 × 4.6 mm i.d., Nacalai, Kyoto, Japan).
